# Impact of Transcranial Direct Current Stimulation on Cognitive Function, Brain Functional Segregation, and Integration in Patients with Mild Cognitive Impairment According to Amyloid-Beta Deposition and *APOE* ε4-Allele: A Pilot Study

**DOI:** 10.3390/brainsci11060772

**Published:** 2021-06-10

**Authors:** Dong-Woo Kang, Sheng-Min Wang, Tae-Yeong Kim, Donghyeon Kim, Hae-Ran Na, Nak-Young Kim, Chang-Uk Lee, Hyun-Kook Lim

**Affiliations:** 1 Department of Psychiatry, Seoul St. Mary’s Hospital, College of Medicine, The Catholic University of Korea, Seoul 06591, Korea; kato7@hanmail.net (D.-W.K.); jihan@catholic.ac.kr (C.-U.L.); 2 Department of Psychiatry, Yeouido St. Mary’s Hospital, College of Medicine, The Catholic University of Korea, Seoul 07345, Korea; smwang11@naver.com (S.-M.W.); haeranna@gmail.com (H.-R.N.); 3 Research Institute, NEUROPHET Inc., Seoul 06247, Korea; ty.kim@neurophet.com (T.-Y.K.); donghyeon.kim@neurophet.com (D.K.); 4 Department of Psychiatry, Keyo Hospital, Uiwang 16062, Korea; nakyoung17@gmail.com

**Keywords:** amyloid beta deposition, *APOE* ε4-allele, mild cognitive impairment, transcranial direct current stimulation

## Abstract

Anodal transcranial direct current stimulation (anodal-tDCS) is known to improve cognition and normalize abnormal network configuration during resting-state functional magnetic resonance imaging (fMRI) in patients with mild cognitive impairment (MCI). We aimed to evaluate the impact of sequential anodal-tDCS on cognitive functions, functional segregation, and integration parameters in patients with MCI, according to high-risk factors for Alzheimer’s disease (AD): amyloid-beta (Aβ) deposition and *APOE* ε4-allele status. In 32 patients with MCI ([^18^ F] flutemetamol-: *n* = 10, [^18^ F] flutemetamol+: *n* = 22; *APOE* ε4-: *n* = 13, *APOE* ε4+: *n* = 19), we delivered anodal-tDCS (2 mA/day, five times/week, for 2 weeks) over the left dorsolateral prefrontal cortex and assessed the neuropsychological test battery and resting-state fMRI measurements before and after 2 weeks stimulation. We observed a non-significant impact of an anodal-tDCS on changes in neuropsychological battery scores between MCI patients with and without high-risk factors of AD, Aβ retention and *APOE* ε4-allele. However, there was a significant difference in brain functional segregation and integration parameters between MCI patients with and without AD high-risk factors. We also found a significant effect of tDCS-by-*APOE* ε4-allele interaction on changes in the functional segregation parameter of the temporal pole. In addition, baseline Aβ deposition significantly associated negatively with change in global functional integrity of hippocampal formation. Anodal-tDCS might help to enhance restorative and compensatory intrinsic functional changes in MCI patients, modulated by the presence of Aβ retention and the *APOE* ε4-allele.

## 1. Introduction

Alzheimer’s disease (AD) is a leading cause of dementia and imposes a marked social and economic burden. Mild cognitive impairment (MCI), a prodromal AD stage, involves subjective and objective decline in cognitive function, but preservation of the independent daily living ability [1]. Since 10–15% of MCI patients convert to dementia annually, various attempts have been made to delay or prevent the transition to dementia at this stage [2]. Although therapeutic attempts, such as cognitive intervention [3], regular physical exercise [4], and dietary intervention have shown some positive results for changes in cognitive function and biomarkers [5], additional evidence is needed for these interventions to be established as an AD prevention strategy. Furthermore, it is often difficult for MCI patients to perform preventive interventions with increased complexity and to maintain consistency for a significant period [6]. Therefore, the importance of an intervention that can be applied in a simple and fixed manner and maintained consistently for a certain period of time is emphasized.

In this regard, noninvasive brain stimulation has been proposed as a potential treatment option in the course of AD [7]. Transcranial direct current stimulation (tDCS), a type of noninvasive brain stimulation, modulates the excitability of cortical neurons depending on the current flow direction [8]. Moreover, tDCS has synaptic after-effects through long-term potentiation and alter oscillatory brain activity and functional connectivity patterns [9]. 

In some previous studies, AD patients showed improvement in the MMSE score [10], recognition memory [11], and global cognitive performance after tDCS was applied [12], while other studies found no significant difference in cognitive function compared with the sham group [13]. In these studies, the dorsolateral prefrontal cortex (DLPFC) has been most frequently targeted, and tDCS was applied in single or multiple sessions. It has been reported that excitatory input in the DLPFC affects memory performances, boosting parietal capacity and playing a compensatory role against a decline in medial-temporal function [14,15]. There is a relative paucity of studies investigating the impact of tDCS on cognitive performance in patients with MCI. Prior research has shown an improvement in word retrieval performance after single-session anodal-tDCS application to the left ventral inferior frontal gyrus of patients with MCI [16]. However, another study found no significant difference in cognitive test battery scores after nine sessions of anodal-tDCS of the left DLPFC in patients with MCI [17].

Resting-state functional MRI (rs-fMRI) reveals intrinsic brain activity in the resting state and can approach functional segregation and integration by evaluating the fractional amplitude of low-frequency fluctuation (fALFF) and degree centrality (DC) [18]. Previous studies have shown that changes and disruptions in functional segregation and integration are associated with AD progression [19,20]. Additionally, the default mode network (DMN) is a characteristic network of increased intrinsic brain activity during the resting state [21], and aberrant changes in this network have been demonstrated to reflect deterioration of AD [22]. Direct current stimulation has been documented to modulate the DMN and affect changes in functional segregation and integration parameters [22,23]. However, few studies have evaluated the impact of tDCS on functional segregation and integration of intrinsic brain activity in the prodromal stage of AD.

Amyloid-beta (Aβ) retention and *APOE* ε4 genotype, which are representative factors affecting the progression and prognosis of AD, have been reported to affect the neuronal activity and cognitive decline significantly [24,25,26,27]. Furthermore, these AD risk factors have been demonstrated to affect the outcomes of preventive attempts in the prodromal stage of AD [28,29]. Nevertheless, few studies have examined the effects of tDCS on cognitive and functional brain changes according to these AD risk factors in the MCI stage and there is little evidence for a precision medicine approach to the tDCS in the prodromal stage of AD.

Consequently, this study evaluated the impact of anodal-tDCS on cognitive performance and functional segregation and integration parameters in MCI patients, depending on Aβ deposition and *APOE* ε4-allele status. We hypothesized that there would be a significant difference in changes in cognitive function and intrinsic brain activity between MCI patients with and without AD risk factors after multiple sessions of anodal-tDCS. Furthermore, we also explored whether the interaction between anodal-tDCS application and AD risk factors affects changes in cognitive function and intrinsic brain activity in the prodromal stage of AD.

## 2. Materials and Methods

### 2.1. Participants

Thirty-two MCI patients were recruited from the Brain Health Center, Yeoui-do St. Mary’s Hospital, College of Medicine, The Catholic University of Korea, Republic of Korea, from May 2020 to December 2020. The study was conducted in accordance with the Declaration of Helsinki and was approved by the Institutional Review Board of the Catholic University of Korea. Informed and written consent was obtained from all participants.

The cognitive functions of all subjects were assessed with the Korean version of the Consortium to Establish a Registry for Alzheimer’s Disease (CERAD-K) [30], which included a verbal fluency (VF) test, the 15-item Boston Naming Test (BNT), the Korean version of the Mini-Mental State Examination (MMSE-K) [31], word list memory (WLM), word list recall (WLR), word list recognition (WLRc), constructional praxis (CP), and constructional recall (CR) assessments. Additionally, total scores of memory domains (TM) were obtained by summing the CERAD-K, WLM, WLR, WLRc, and CR scores. Total CERAD-K scores were calculated by summing all CERAD-K subcategory scores, excluding the MMSE-K score. 

Patients with amnestic MCI met Peterson’s criteria [32]. Inclusion criteria for MCI participants are described in a detail in the Supplementary Material. All subjects were evaluated at the Brain Health Center by an experienced psychiatrist and a psychologist. Details surrounding the usage of specific tests and the reviewing process are described in the Supplementary Material. 

### 2.2. Experimental Design

In this study, patients received 10 tDCS sessions (five times/week for 2 weeks: 10 sessions). The participants were assessed with the CERAD-K neuropsychological battery and underwent resting-state fMRI within 2 weeks before the first tDCS session and after the 10 th session. Subjects underwent [^18^ F] flutemetamol (FMM) positron emission tomography‒computed tomography (PET-CT) and *APOE* genotyping within 4 weeks before the first tDCS session. In addition, participants and the psychologists who performed the neuropsychological battery were blinded to the results of amyloid-PET and *APOE* genotyping.

### 2.3. tDCS Application

A constant direct current (2 mA, 20 min) was administered by an MRI-compatible stimulator (YDS-301N, YBrain, Seoul, Republic of Korea). The anode was attached over the left DLPFC (F3 in the International 10/20 electroencephalogram system). The cathode was positioned over the right supraorbital region. The electrodes touched a water-soaked sponge (disc type, radius = 3 cm) placed on the scalp. For the subject to apply the device accurately, staff skilled in the use of the device visited the patient’s residence for each stimulus session to guide device application. 

### 2.4. fMRI Data Acquisition and Data Processing

Imaging data were collected by the Department of Radiology of Yeouido Saint Mary’s Hospital at the Catholic University of Korea using a 3-T Siemens Skyra MRI machine and a 32-channel Siemens head coil (Siemens Medical Solutions, Erlangen, Germany). Parameters of structural and functional MRI data acquisition are described in the Supplementary Material. 

We used the Data Processing Assistant for Resting-State fMRI (DPARSF, GNU GENERAL PUBLIC LICENSE, Beijing, China) [33], which is based on Statistical Parametric Mapping (SPM, http://www.fil.ion.ucl.ac.uk/spm, Wellcome Centre for Human Neuroimaging, London, UK), to preprocess the fMR images. Slice timing and realignment for motion corrections were performed on the images. Subjects with excessive head motion (cumulative translation or rotation > 2 mm or 2°) were excluded. To prevent group-related differences caused by microscopic head motion, framewise displacement (FD) was compared between the groups. Mean FD scores did not differ between groups (*p* > 0.05, 2-sample *t* test). For spatial registration, T1-weighted images were co-registered to the mean rsfMRI image based on rigid-body transformation. For spatial normalization, the International Consortium for Brain Mapping template was applied (resampling voxel size = 3 × 3 × 3 mm) and fitted to the “East Asian brain”. After this, the functional images were spatially smoothed with a 6 mm full width at half maximum Gaussian kernel. 

We further processed our functional data to fit them to fALFF and DC analysis with DPARSF. Linear trends were removed from the functional images, and data were filtered with a temporal band-pass of 0.01–0.08 Hz, to reduce low-frequency drift as well as physiological high-frequency respiratory and cardiac noise. Several nuisance covariates were regressed out, including six head motion parameters and signals from the WM and CSF.

### 2.5. fALFF and DC Analysis

To measure regional intrinsic brain activities in the resting state, fALFF was computed using individual preprocessed data [19]. The process of calculating fALFF is described in detail in the Supplementary Material. This fALFF calculation was repeated for each voxel in the whole brain to create a fALFF map for each participant, which was used in statistical analysis.

The DC was computed as the number of significant correlations (binarized) or as the sum of the weights of the significant connections (weighted) for each voxel (a threshold of r > 0.25, *p* < 0.05). The map of the connectivity was then standardized by conversion to z scores, so that maps across participants could be averaged and compared. DC represents the most local and directly quantifiable centrality measure and has been widely used to examine node characteristics of intrinsic network connectivity [34]. Within the DMN, the DC value of a node indicates its connectivity strength to all the other nodes and reflects its importance in functional integration. Additionally, the fALFF and DC were calculated in 11 predefined regions-of-interest (ROIs) in the DMN and were used in statistical analysis (Table S1 in the Supplementary Materials) [35]. Moreover, whole-brain voxel-wise analysis of fALFF and DC was also performed. 

### 2.6. [^18^ F] Flutemetamol PET-CT Image Acquisition, Assessments, and SUVR Calculations 

[^18^ F] FMM was manufactured, and FMM-PET data were collected and analyzed as described previously [36]. MRI of each participant was used to co-register and define the ROIs, and correct partial volume effects arising from the expansion of cerebrospinal spaces accompanying cerebral atrophy. We used a standardized uptake value ratio (SUVR) at 90-min post-injection to analyze the FMM PET data, using the pons ROI as the reference. Global Aβ burden was expressed as the average SUVR of the mean for the six cortical ROIs, including the frontal, superior parietal, lateral temporal, striatum, anterior, and posterior cingulate cortex/precuneus ROIs. We used a cut-off for “high” or ‘low’ neocortical SUVR of 0.62, consistent with cut-off values used in previous FMM PET study [36].

### 2.7. Statistical Analysis

Statistical analyses for demographic data were performed using R software (version 2.15.3). Assumptions of normality were tested for continuous variables using the Kolmogorov–Smirnov test; all data demonstrated a normal distribution. Two sample t-tests and chi-square (χ^2^) tests were used to probe for differences in demographic variables, clinical data, cognitive function, and fMRI measurements between MCI patients with and without Aβ deposition and the *APOE* ε4-allele. Cognitive function and fMRI parameters (fALFF and DC in ROIs of the DMN) over 10 sessions were analyzed for change with a repeated-measures analysis of variance (ANOVA) with time (pre-tDCS and post-tDCS) as repeated-measures factor and the presence of Aβ deposits and the *APOE* ε4-allele as the between-subject factor, with adjustments for age, sex, and years of education. Multiple regression analysis was performed to evaluate the association between baseline [^18^ F] FMM SUVR_PONS_ and change in cognitive function and rs-fMRI measurements (fALFF and DC in ROIs of DMN), adjusting for age, sex, education years, and *APOE* ε4-allele. Each variable was z-transformed using the mean and standard deviation. All statistical analyses used a two-tailed *p*-value < 0.05 to define statistical significance. 

Additionally, to observe the effects of tDCS-by-group interaction on fALFF and DC, a mixed analysis on a voxel-by-voxel basis, with groups (*APOE* ε4-allele carrier vs. non-carrier; positive vs. negative for Aβ retention) as between-subject factors and tDCS (pre-tDCS vs. post-tDCS) as within-subject factors was performed on a brain mask. Age, sex, and years of education were included as covariates in the statistical tests. We designed a mixed analysis based on the SPM 12. An F-contrast was designed for the interaction effect analysis. Furthermore, paired t-tests were performed between pre-tDCS and post-tDCS on the individual z maps of fALFF and DC in each sub-group, respectively (negative or positive for Aβ retention; *APOE* ε4-allele carrier or non-carrier). All statistical maps were corrected for multiple comparisons by Gaussian random field (GRF) correction combining the voxel *p*-value < 0.001 and cluster level < 0.05 in DPABI_V5.1_201201 (http://rfmri.org/dpabi, GNU GENERAL PUBLIC LICENSE, Beijing, China) [37].

## 3. Results

### 3.1. Baseline Demographic and Clinical Data

Table 1 shows the baseline demographic and clinical data for MCI patients classified by the presence of Aβ deposits and the *APOE* ε4-allele. MCI patients with Aβ deposits showed higher years of education than those without Aβ accumulation (Table 1A). The ratio of *APOE* ε4 carriers was significantly higher in the group with Aβ deposits. This group displayed higher average SUVR_PONS_ than that without Aβ deposits (Table 1A). 

There were no significant differences in age, sex, and years of education between patients with MCI with and without the *APOE* ε4-allele (Table 1B). We found a higher ratio of Aβ deposits in *APOE* ε4 carriers. *APOE* ε4 carriers showed higher average SUVR_PONS_ than non-carriers (Table 1B).

### 3.2. Changes in Neuropsychological Performance 

For the CERAD-K subdomain and total scores, after adjustment for age, sex, and years of education, the main effect for the tDCS, Aβ deposits, and *APOE* genotype was not significant. Additionally, there was a nonsignificant interaction between tDCS and AD risk factors, including *APOE* genotype and Aβ deposition. 

### 3.3. Changes in Functional Segregation and Integration of the DMN: An ROI-Based Analysis

**Figure 1:**
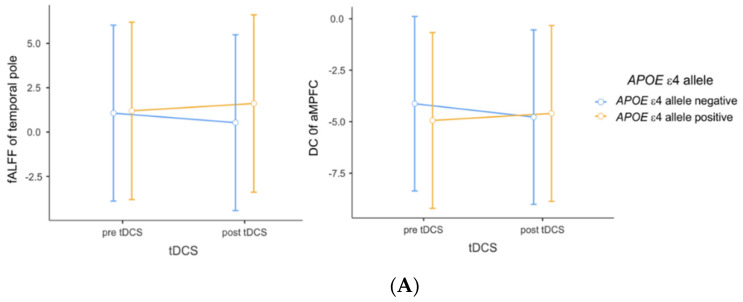

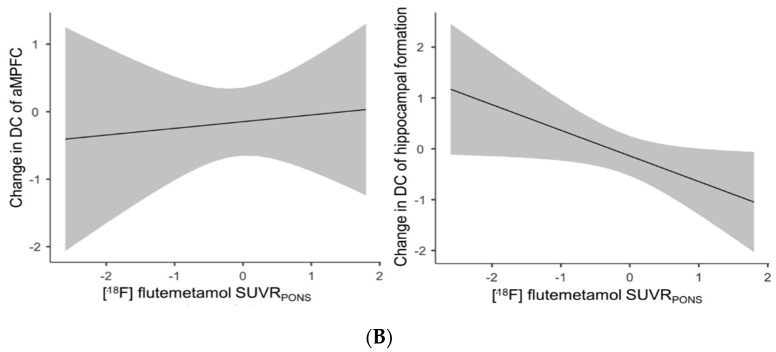
(**A**) Impact of interaction between tDCS and *APOE* ε4 allele on fALFF and DC. (**B**) Associations between [^18^ F] flutemetamol SUVR_PONS_ and changes in amplitude of DC. Repeated-measures analysis of variance was used to predict the impact of interaction (**A**) between tDCS and *APOE* ε4 allele on fALFF and DC, with adjustment for age, sex, and education years (*p* = 0.036, partial η^2^ = 0.164; *p* = 0.056, partial η^2^ = 0.138, respectively). (**B**) Multiple linear regression analysis was used to evaluate the associations between [^18^ F] flutemetamol SUVR_PONS_ and changes in DC before and after tDCS, with adjustment for age, sex, and education years (*p* = 0.075, R^2^ = 0.240; *p* = 0.042, R^2^ = 0.182). Each variable was z-transformed using the mean and standard deviation. Changes in fALFF and DC were defined as post-tDCS z-transformed values minus pre-tDCS z-transformed values. Abbreviations: tDCS, transcranial direct current stimulation; fALFF, fractional amplitude of low-frequency fluctuation; DC, degree centrality; SUVR, standardized uptake value ratio; Aβ, amyloid beta; aMPFC, anterior medial prefrontal cortex.

In terms of functional segregation of the DMN, the main effects of tDCS, Aβ deposits, and *APOE* genotype were not significant. Additionally, there was nonsignificant interaction between tDCS and Aβ deposition. However, there was an interaction between tDCS and the *APOE* ε4-allele, which could be attributed to increased temporal pole fALFF after tDCS application in MCI *APOE* ε4-allele carriers (*p* = 0.036, Figure 1A). Additionally, this interaction yielded a large effect size (partial η^2^ = 0.164). However, we found a nonsignificant association between the baseline average SUVR_PONS_ and change in temporal pole fALFF (*p* = 0.090). Despite not reaching statistical significance, this association showed a large effect size (R^2^ = 0.289).

Regarding functional integration of the DMN, the main effect of tDCS and AD risk factors was not significant. Although as well as a nonsignificant interaction between tDCS and AD risk factors, there was a statistical trend toward an interaction between tDCS and the *APOE* ε4-allele, possibly attributable to increased aMPFC DC after tDCS application in MCI patients with Aβ deposits, yielding a large effect size (*p* = 0.056, partial η^2^ = 0.138, Figure 1A). Additionally, we found a statistical trend toward a positive association between baseline average SUVR_PONS_ and change in aMPFC DC (*p* = 0.075, R^2^ = 0.240, Figure 1B), but a negative association between average SUVR_PONS_ and change in hippocampal formation DC (*p* = 0.042, R^2^ = 0.182, Figure 1B), with a large effect size.

### 3.4. Changes in Functional Segregation and Integration Parameters: Whole Brain Voxel-Based Analysis

**Figure 2:**
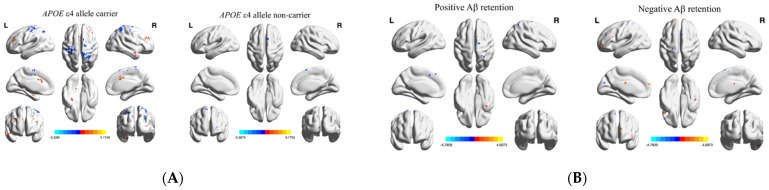
Significant changes in fALFF after tDCS for patients with mild cognitive impairment (**A**) with and without the *APOE* ε4 allele, and (**B**) with and without Aβ deposits. Whole-brain voxel-wise fALFF analysis results. Thresholds were set using GRF correction at a *p*-value of <0.05, voxel *p* < 0.001. The statistical threshold of the cluster size is described in Figure 2. Abbreviations: tDCS, transcranial direct current stimulation; DC, degree centrality; Aβ, amyloid beta.

No brain regions showed a significant impact of tDCS-by-group interaction on the fALFF and DC in each sub-group. Brain regions that showed changes in fALFF after tDCS according to *APOE* genotype and Aβ deposition are displayed in Figure 2A,B. The brain regions that showed significant changes in fALFF differed between MCI *APOE* ε4 carriers and non-carriers. Additionally, increased and decreased fALFF values were observed in the right inferior temporal gyrus and crus I of the cerebellum, respectively, after tDCS, in both MCI patients with and without Aβ deposition. However, other brain regions that showed significant changes in fALFF also differed between MCI patients with and without Aβ deposits.

**Figure 3:**
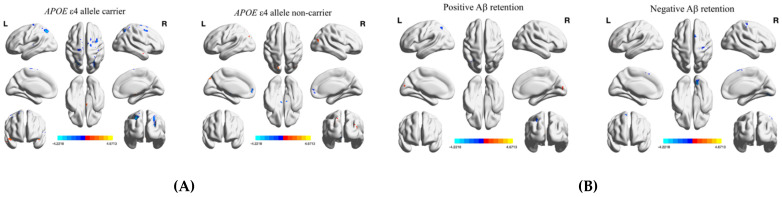
Significant changes in DC after tDCS of mild cognitive impairment patients (**A**) with and without the *APOE* ε4 allele, and (**B**) with and without Aβ deposits. Whole-brain voxel-wise fALFF analysis results. Whole-brain voxel-wise DC analysis results. Thresholds were set using GRF correction at a p-value of <0.05, voxel *p* < 0.001. The statistical threshold of the cluster size is described in Figure 3. Abbreviations: MNI, Montreal Neurological Institute coordinate; aMCI, amnestic mild cognitive impairment group; L/R, left/right; tDCS, transcranial direct current stimulation; fALFF, fractional amplitude of low-frequency fluctuation.

In terms of functional integration, brain regions that showed changes in DC after tDCS according to *APOE* genotype and Aβ deposition are shown in Figure 3A,B. The brain regions that showed significant changes in DC differed between MCI APOE4 carriers and non-carriers and patients with and without Aβ deposits. These anatomical regions, their corresponding MNI coordinates, and the intensity of peak points in each cluster are shown in Table 2 and Table 3.

## 4. Discussion

The current study aimed to evaluate the impact of anodal-tDCS on cognitive performance and functional segregation and integration parameters in MCI patients, according to the presence of Aβ deposits and the *APOE* ε4-allele. We evaluated the effect of interactions between anodal-tDCS application and AD risk factors on changes in cognitive function and intrinsic brain activity and explored differences in changes in cognitive function and spontaneous brain activity parameters between MCI patients with and without AD risk factors after multiple sequential anodal-tDCS sessions.

### 4.1. Changes in Neuropsychological Performance

With regard to cognitive performance, the impact of tDCS was not significant for changes in cognitive performance. We also found a nonsignificant interaction between anodal-tDCS and AD risk factors in the current study.

Similarly, in a previous study that conducted a nine-session clinical trial for 3 weeks in MCI patients, there was no improvement in the objective neuropsychological test score [17]. On the other hand, some prior studies have demonstrated improvement of semantic word-retrieval performance after a single-session anodal-tDCS application over the left ventral inferior frontal gyrus of MCI patients [16]. In previous studies that performed anodal-tDCS on AD patients, they reported improved MMSE scores [10], recognition memory [11], and global functioning as compared to the sham group [12]. Additionally, in a meta-analysis of administering tDCS in patients with mild to moderate AD, repeated-session tDCS was not significantly more effective than single-session tDCS [38]. Moreover, stimulation of the temporal cortex significantly improved cognitive function, as compared to other areas, although the left DLPFC was the most frequently stimulated area [38]. The tDCS protocol of the present study did not contain factors that show beneficial effects identified in the meta-analysis, which could contribute to the restricted improvement in cognitive function. However, this meta-analysis targeted only seven studies, and the sample size was small, and thus results should be interpreted cautiously. Additionally, the insufficient sample size of the current pilot study could contribute to the nonsignificant change in cognitive function.

### 4.2. Changes in Functional Segregation and Integration of the DMN: An ROI-Based Analysis

With regard to changes in brain functional segregation parameters, this study found a significant interaction between tDCS and *APOE* ε4-allele in the left temporal pole. This interaction could contribute to increased temporal pole fALFF after anodal-tDCS application in MCI patients. The left temporal pole is part of the DMPFC subsystem of the DMN, which is vulnerable to AD pathology [35]. The DC of the left temporal lobe is lower in patients with MCI than in cognitively intact older adults [39]. Additionally, the temporal pole was associated with an abnormal insula network in MCI patients, and decreased functional connectivity in this network is related to cognitive decline in MCI patients [40]. Furthermore, the *APOE* ε4-allele reduces connectivity of the hippocampal network, which includes the temporal pole in healthy older adults [26]. Although the present study showed a relative lack of evidence for functional integration changes, application of anodal-tDCS in prodromal AD patients with high-risk factors appears to restore the local intrinsic change in the temporal pole found in the MCI stage. This observation might support the hypothesis that tDCS-induced improvement is related to the restoration, rather than compensation, of brain activity patterns [41].

In this study, although there was a lack of a statistical significance, the index reflecting the global functional integration of aMPFC also showed a similar pattern to the interaction found in the functional segregation parameter of the temporal pole, yielding a large effect size. These results might be attributed to increased functional integration after anodal-tDCS application in MCI patients with the *APOE* ε4 genotype. The aMPFC is an anterior core set of hubs in the DMN and shows global connectivity with other areas that constitute a DMN subsystem [35]. Additionally, the anterior DMN shows increased connectivity during AD and cognitive decline progression, and this change in the anterior hubs may be a compensatory response to AD pathology [42]. However, it is possible that these results may underestimate Aβ-mediated hyperactivation in the early stages of AD [43]. Therefore, it is important to bear in mind the possible bias in these responses.

Another important finding was that a decreased change in DC of hippocampal formation was exhibited in the higher baseline Aβ deposits with a large effect size. This result might reflect decoupling of the hippocampal formation from posterior DMN nodes at the prodromal AD stage [44], and it is estimated that the tDCS application does not significantly affect pathologic functional changes in the hippocampal formation.

### 4.3. Changes in Functional Segregation and Integration Parameters: Whole Brain Voxel-Based Analysis

Lastly, in the present study, differences were observed in changing functional segregation and integration patterns after anodal-tDCS application, depending on the *APOE* ε4-allele or Aβ deposits by whole-brain voxel-based analysis in MCI patients. In terms of functional segregation parameters after anodal-tDCS application, our MCI patients with *APOE* ε4-allele displayed increased local intrinsic brain activity in DMN hub regions and AD compensatory regions, in which previous studies have shown a decreasing trend of fALFF across the AD spectrum [45]. However, MCI patients without the *APOE* ε4-allele showed increased fALFF after repetitive anodal-tDCS administration in different brain regions, such as the inferior occipital gyrus, calcarine fissure, and surrounding cortex. The inferior occipital gyrus has been documented to be vulnerable during the MCI stage and is connected with deep brain structures related to MCI pathology [46]. Additionally, the fALFF of the calcarine fissure and surrounding cortex showed a decreasing trend during the AD course [47]. However, the lack of information on the *APOE* genotype in previous reports adds further caution regarding the interpretation of these findings. In MCI patients in the present study, regional intrinsic activity of the inferior temporal gyrus was increased both with and without Aβ deposits, and this region has shown lower local integrity in the MCI group than in the normal group in our previous study [48]. Furthermore, the cerebellum, in which regional intrinsic brain activity increased after tDCS in MCI patients with Aβ deposits, was also the area in which fALFF tended to decrease with AD progression in a previous study [45]. Therefore, these findings might indicate that increased fALFF in functionally deteriorated regions might be induced by sequential anodal-tDCS during the prodromal AD stage. Additionally, MCI patients without Aβ deposits showed increased intrinsic brain activity at various locations in the frontal gyrus, unlike those with Aβ deposition after multiple sessions of anodal-tDCS. In a prior study, the frontal cortex showed hypermetabolism in MCI patients without Aβ accumulation, and MCI patients with cortical hypermetabolism did not convert to AD during the follow-up period [49]. Hence, it could conceivably be hypothesized that sequential anodal-tDCS may restore spontaneous brain activity in MCI patients with Aβ deposits but play a compensatory role in those without Aβ deposition. Future studies on the current topic are therefore recommended.

Regarding the functional integration parameter evaluated by whole-brain voxel-based analysis, we found that MCI patients with AD risk factors showed increased DC in the cerebellum after anodal-tDCS, similar to the pattern of functional segregation parameter changes. Another finding was that MCI patients with the *APOE* ε4-allele showed increased temporal pole DC after anodal-tDCS, in which a fALFF increase was observed in ROI-based analysis. According to these data, it might be assumed that the intensity at which a region locally activated by anodal-tDCS is integrated with other regions increases simultaneously in MCI patients with high-risk factors of AD.

### 4.4. Limitations

A significant limitation of the current pilot study is that the sample size was relatively small, and no comparisons with a sham group were made. Consequently, there is a relative lack of statistical robustness for the interaction between anodal-tDCS application and AD risk factors for changes in cognitive function and brain functional segregation and integration. Lastly, considering the after-effects of tDCS [9] and the important role of stimulation frequency for outcomes in MCI and AD patients [50], further research, applying tDCS for a longer duration, is needed.

## 5. Conclusions

This study provides an initial step in searching for conditions that may deliver optimal effects when tDCS is administered during the AD prodromal stage. It is necessary to identify the preventive and therapeutic mechanisms of tDCS in AD more clearly, and to establish a foundation for precision medicine for tDCS treatment of AD.

## Figures and Tables

**Table 1:** Demographic and clinical characteristics of study participants categorized by (**A**) Aβ deposits and (**B**) *APOE* ε4 allele.

(**A**)			
**Aβ-Deposits**	**Aβ-Negative**	**Aβ-Positive**	***p***
	(N = 10)	(N = 22)	
Age	77.5 ± 6.1	72.3 ± 7.1	0.054
Sex			>0.999
Male	4 (40.0%)	9 (40.9%)	
Female	6 (60.0%)	13 (59.1%)	
Years of education	9.6 ± 4.4	13.5 ± 4.9	0.039
*APOE* ε4 allele			0.001
Non-carrier	9 (90.0%)	4 (18.2%)	
Carrier	1 (10.0%)	18 (81.8%)	
Average SUVR_PONS_ of [^18^ F] flutemetamol	0.5 ± 0.1	0.7 ± 0.1	<0.001
(**B**)			
***APOE* ε4 Allele**	**Non-Carrier**	**Carrier**	***p***
	(N = 13)	(N = 19)	
Age (years)	75.8 ± 6.4	72.6 ± 7.6	0.229
Sex			0.873
Male	6 (46.2%)	7 (36.8%)	
Female	7 (53.8%)	12 (63.2%)	
Years of education	11.1 ± 4.2	13.1 ± 5.5	0.268
Aβ deposits			0.001
Aβ neg	9 (69.2%)	1 (5.3%)	
Aβ pos	4 (30.8%)	18 (94.7%)	
Average SUVR_PONS_ of [^18^ F] flutemetamol	0.6 ± 0.2	0.7 ± 0.1	0.003

Data are presented as the mean ± SD unless indicated otherwise. Aβ Neg, negative deposits of Aβ; Aβ pos, positive deposits of Aβ; SUVR_PONS_, standardized uptake value ratios of [^18^ F] flutemetamol, using pons as a reference region.

**Table 2:** Changes in fALFF of MCI patients after tDCS, according to (**A**) *APOE* genotype and (**B**) Aβ deposits.

(**A**)						
**Region**	**L/R**	**Cluster**	**Peak** **T Value**	**Peak MNI Coordinates** **(x, y, z)**		
**Changes in fALFF of MCI *APOE* ε4 Carriers**						
tDCS > baseline						
Middle temporal gyrus	R	98	3.9585	51	−3	−24
Lobule III of cerebellum	L	187	5.0774	−3	−45	−21
Parahippocampal gyrus	R	40	3.2239	33	−18	−30
Precuneus	L	41	4.831	−18	−48	3
Inferior frontal gyrus, triangular part	L	81	3.4026	−45	30	24
Middle cingulate gyri	R	62	3.8016	6	12	36
Midcingulate area	L	189	3.1684	−12	−42	51
tDCS < baseline						
Middle occipital gyrus	R	45	−5.1376	27	−75	30
Superior frontal gyrus	L	384	−5.2261	−30	−3	69
Superior parietal gyrus	R	250	−4.3472	27	−51	72
Postcentral gyrus	L	38	−4.5553	−42	−21	57
Supplementary motor area	R	43	−3.7324	3	18	63
Superior frontal gyrus	R	61	−4.9716	15	−9	69
**Changes in fALFF of MCI *APOE* ε4 Non-Carriers**						
tDCS > baseline						
Inferior occipital gyrus	L	29	5.1353	−42	−72	−6
Calcarine fissure and surrounding cortex	R	29	3.8547	18	−81	9
tDCS < baseline						
Superior frontal gyrus, orbital	L	47	−4.0932	−27	57	−3
Supplementary motor area	L	79	−4.4351	−3	15	63
(**B**)						
**Region**	**L/R**	**Cluster**	**Peak** **T Value**	**Peak MNI Coordinates** **(x, y, z)**		
**Changes in fALFF of MCI Patients with Aβ Deposits**						
tDCS > baseline						
Inferior temporal gyrus	R	61	4.0777	39	−6	−45
Crus I of of cerebellum	L	36	4.6073	−15	−72	−33
Lobule III of cerebellum	L	62	4.374	−3	−45	−21
tDCS < baseline						
Crus I of of cerebellum	R	40	−4.5424	36	−81	−36
Supramarginal gyrus	R	38	−3.4884	60	−30	33
Superior parietal gyrus	R	99	−4.3392	27	−51	72
Superior frontal gyrus, medial	R	146	−3.5363	9	33	57
Superior frontal gyrus	L	119	−4.3879	−27	−9	72
Paracentral lobule	L	35	−2.7845	−6	−30	78
**Changes in fALFF of MCI Patients without Aβ Deposits**						
tDCS > baseline						
Inferior temporal gyrus	R	29	4.9944	48	−21	−27
Middle temporal gyrus	L	28	6.7989	−57	−36	−6
Middle frontal gyrus	L	42	6.345	−39	45	−9
Inferior frontal gyrus triangular part	L	32	5.266	−42	18	6
Superior frontal gyrus, medial	L	27	7.7112	−9	63	21
Precuneus	R	32	4.4101	9	−60	54
tDCS < baseline						
Crus I of cerebellum	R	27	−4.3276	48	−54	−27
Cuneus	L	36	−4.2645	−6	−81	24
Superior occipital gyrus	R	30	−4.1793	24	−90	27
Middle frontal gyrus	R	29	−5.2138	45	12	42
Supplementary motor area	R	27	−6.8382	6	15	63
Postcentral gyrus	L	28	−3.587	−24	−27	72

Thresholds were set using GRF correction at a p-value of <0.05, voxel *p* < 0.001. (**A**) *APOE* ε4 allele carrier, cluster size >38; *APOE* ε4 allele non-carrier, cluster size > 29; (**B**) Aβ deposit-positive, cluster size >35; Aβ deposit-negative, cluster size > 27. Brain regions that showed significant changes are described in Table 2. Abbreviations: tDCS, transcranial direct current stimulation; fALFF, fractional amplitude of low-frequency fluctuation; Aβ, amyloid beta.

**Table 3:** Changes in DC of MCI patients after tDCS, according to (**A**) *APOE* genotype and (**B**) Aβ deposits.

(**A**)						
**Region**	**L/R**	**Cluster**	**Peak** **T Value**	**Peak MNI Coordinates** **(x, y, z)**		
**Changes in DC of MCI Patients with *APOE* ε4 Carrier**						
tDCS > baseline						
Lobule VIIB of cerebellar hemisphere	R	78	3.7442	30	−72	−48
Temporal pole: superior temporal gyrus	R	115	4.3302	45	3	−15
Temporal pole: superior temporal gyrus	L	108	4.3954	−42	3	−15
Calcarine fissure and surrounding cortex	R	67	3.7306	9	−87	9
tDCS < baseline						
Superior parietal gyrus	R	178	−4.1919	27	−63	51
Superior parietal gyrus	L	767	−5.1001	−24	−69	48
**Changes in DC of MCI Patients with *APOE* ε4 Non-Carrier**						
tDCS > baseline						
Inferior temporal gyrus	L	48	4.5971	−42	−60	−6
Middle occipital gyrus	R	60	4.2846	33	−78	30
Middle occipital gyrus	L	84	4.7948	−15	−81	39
tDCS < baseline						
Superior frontal gyrus, medial	L	338	−6.6552	0	57	0
(**B**)						
**Region**	**L/R**	**Cluster**	**Peak** **T Value**	**Peak MNI Coordinates** **(x, y, z)**		
**Changes in DC of MCI Patients with Aβ Deposits**						
tDCS > baseline						
Lobule VIII of cerebellum	R	70	3.9953	12	−66	−45
Calcarine fissure and surrounding cortex	R	230	4.6267	9	−87	9
tDCS < baseline						
Superior parietal gyrus	L	76	−3.427	−21	−51	45
**Changes in DC of MCI Patients without Aβ Deposits**						
tDCS > baseline						
Superior frontal gyrus, medial orbital	R	53	4.7797	12	66	−9
Middle frontal gyrus	L	70	4.8997	−48	42	15
Middle temporal gyrus	R	53	4.9548	66	−48	9
tDCS < baseline						
Lingual gyrus	R	55	−5.8024	12	−81	−12
Superior frontal gyrus, medial	L	48	−4.093	0	57	0
Lenticular nucleus, Putamen	L	48	−4.2643	−18	3	6
Middle occipital gyrus	L	50	−3.9537	−24	−84	9
Supplementary motor area	R	115	−3.9209	12	12	69
Precentral gyrus	L	59	−3.5792	−33	−21	72
Postcentral gyrus	R	153	−5.5196	27	−30	60

Whole-brain voxel-wise DC analysis results. Thresholds were set using GRF correction at a *p*-value of <0.05, voxel *p* < 0.001. (**A**) *APOE* ε4 allele carrier, cluster size > 62; *APOE* ε4 allele non-carrier, cluster size > 48; (**B**) Aβ deposit-positive, cluster size > 52; Aβ deposit-negative, cluster size > 48. Brain regions that showed significant changes are described in Table 3. Abbreviations: MNI, Montreal Neurological Institute coordinate; aMCI, amnestic mild cognitive impairment group; L/R, left/right; tDCS, transcranial direct current stimulation; fALFF, fractional amplitude of low-frequency fluctuation.

## Data Availability

The datasets generated or analyzed during the current study are not publicly available due to Patient Data Management Protocol of Yeouido St. Mary’s Hospital but are available from the corresponding author on reasonable request.

## Supplementary Materials

The following are available online at https://www.mdpi.com/article/10.3390/brainsci11060772/s1, Table S1: Regions-of-interest (ROIs) in the default network.
