# Supplementary material for: Impact of Transcranial Direct Current Stimulation on Cognitive Function, Brain Functional Segregation, and Integration in Patients with Mild Cognitive Impairment According to Amyloid-Beta Deposition and APOE ε4-Allele: A Pilot Study
Source: Brain Sci. 2021 Jun 10;11(6):772. doi: 10.3390/brainsci11060772 (PMC8230518; doi:10.3390/brainsci11060772)
Supplement: Supplementary file 1 [file brainsci-11-00772-s001.zip › brainsci-1242199-supplementary.pdf]

brain sciences

MDPI

Supplementary Material

Impact of transcranial direct current stimulation on cognitive function, brain functional segregation, and integration in patients with mild cognitive impairment according to amyloid-beta deposition and *APOE* ε4-allele: A pilot study

Dong-Woo Kang [1], Sheng-Min Wang [2], TaeYeong Kim [3], Donghyeon Kim [3], Hae-Ran Na [2], Nak-Young Kim [4], Chang-Uk Lee [1], and Hyun Kook Lim [2,*]

[1] Department of Psychiatry, Seoul St. Mary's Hospital, College of Medicine, The Catholic University of Korea, Seoul 06591, Korea; kato7@hanmail.net (D.W.K.); ji-han@catholic.ac.kr (C.U.L.)
[2] Department of Psychiatry, Yeouido St. Mary's Hospital, College of Medicine, The Catholic University of Korea, Seoul 07345, Korea; smwang11@naver.com (S.-M.W.); haeranna@gmail.com (H.-R.N.)
[3] Research Institute, NEUROPHET Inc.; Seoul 06247, Korea; ty.kim@neurophet.com (T.K.); donghyeon.kim@neurophet.com (D.K.)
[4] Department of Psychiatry, Keyo Hospital, Uiwang 16062, Korea; nakyoung17@gmail.com (N.−Y.K.)
* Correspondence: drblues@catholic.ac.kr (H.K.L.)

1 Supplementary Methods

1.1 Neuropsychological evaluation

Cognitive status was assessed by neuropsychological testing at Seoul St. Mary's Hospital, The Catholic University of Korea. The cognitive functions of all the subjects were assessed with the Korean version of the Consortium to Establish a Registry for Alzheimer's Disease (CERAD-K),

which included Verbal Fluency (VF), the 15-item Boston Naming Test (BNT), MMSE-K, Word List Memory (WLM), Word List Recall (WLR), Word List Recognition (WLRc), Constructional Praxis (CP), and Constructional Recall (CR). The CERAD is the standardized clinical and neuropsychological assessment battery for the evaluation of patients with Alzheimer's disease. The results were reviewed by a neuropsychologist to determine whether there was evidence of cognitive impairment.

The VF score is the number of animal names that the subject could name in one minute. The BNT score ranges from 0 to 15 points. The MMSE-K score ranges from 0 to 30 points. The WLM score ranges from 0 to 30 points. The WLR score ranges from 0 to 10 points. The WLR score ranges from 0 to 10 points. The WLRc score ranges from 0 to 10 points. The CP score ranges from 0 to 11 points. The CR score ranges from 0 to 11 points.

1.2 *APOE* genotyping

DNA was isolated from blood using the QIAmp Blood DNA Maxi Kit protocol (Qiagen, Valencia, CA). Genotypes for two APOE SNPs, rs429358 (E*4) and rs7412 (E*2) were determined using TaqMan SNP genotyping assays (Applied Biosystems, Foster City, California).

1.3 Inclusion and exclusion criteria for participants

Inclusion criteria were as follows: patients with MCI met Peterson's criteria of (1) memory complaint, corroborated by an informant, (2) objective memory impairment for age, level of education, and sex; (3) essentially preserved general cognitive function; (4) mostly intact functional activities; and (5) no dementia. All patients with MCI had an overall Clinical Dementia Rating of 0.5. Objective memory impairment was defined as a performance score of 1.5 standard deviations (SDs) below the respective age-, education-, and sex-specific normative means on at least one of the four episodic memory tests included in the CERAD-K, that is, WLM, WLR, WLRc, and CR.

We excluded participants with any history of alcoholism, drug abuse, head trauma, or psychiatric disorders, those taking any psychotropic medications (e.g., cholinesterase inhibitors, antidepressants, benzodiazepines, and antipsychotics), those with multiple vascular risk factors, and those with extensive cerebrovascular disease.

1.4 Structural and functional MRI data acquisition

The parameters used for the T1-weighted volumetric magnetization-prepared rapid gradient echo scan sequences were TE =2.6 ms, TR =1,940 ms, inversion time =979 ms, FOV =230 mm, matrix =256×256, and voxel size =1.0×1.0×1.0 mm^3. Resting-state fMR images were collected using a T2* weighting gradient echo sequence with TR = 2,000 ms, TE = 30 ms, matrix = 128 × 128 × 29, and voxel size = 1 × 1 × 2 mm^3. We acquired 150 volumes in 5 minutes, with the instruction, "keep your eyes closed and think of nothing in particular."

1.5 The process of calculating fALFF

fALFF is the ratio between the sum of the Fourier amplitudes within a specific low-frequency range (0.01–0.1 Hz) and the sum of the Fourier amplitudes across the entire frequency range (0–0.2 Hz). Fast Fourier transform (FFT) was applied to transform the time series of each voxel to the frequency domain and obtain the power spectrum. The power

spectrum obtained by FFT was square-rooted and then averaged across 0.01–0.08 Hz at each voxel, which is defined as ALFF. The fraction of ALFF in a given frequency band to ALFF over the entire frequency range yielded fALFF values.

1.6 [^{18}F] flutemetamol PET image assessments & SUVR calculation

1.6.1 PET scanners

Each scanner was commissioned by scanning a NEMA phantom and adjusting the reconstruction parameters to obtain a spatial resolution of ~6.5mm. This optimization was performed in advance of the scanning of patients and the images received by GE were not subject to any further post-processing in regards of spatial resolution.

1.6.2 SUVR calculation

The [^{18}F] flutemetamol (FMM) standardized uptake values (SUVs) were measured for six volumes of interest (VOIs) restricted to gray matter and adjusted for atrophy manually, covering the frontal, superior parietal, lateral temporal, striatum, anterior, and posterior cingulate cortex/precuneus ROIs. These VOIs are reference areas used in visual assessment of [^{18}F] FMM PET images [1]. Frame-to-frame motion correction was performed on the dynamic data before quantitative SUV measurements, and standard uptake value ratio (SUVR) calculations were made. We obtained an SUV for the pons as a reference. A regional SUVR was calculated as a ratio of each cortical region SUV in VOI to the pons SUV ($SUVR_{PONS}$). A global cortical average (composite SUVR) was calculated, averaging the regional cortical SUVRs.

1.7 Table S1 Regions-of-interest (ROIs) in the default mode network

Brain region	MNI coordinates		
	x	y	z
**PCC-aMPFC Core**			
Anterior medial prefrontal cortex	-6	52	-2
Posterior cingulate cortex	-8	-56	26
**dMPFC Subsystem**			
Dorsal medial prefrontal cortex	0	52	26
Temporal parietal junction	-54	-54	28
Lateral temporal cortex	-60	-24	-18
Temporal pole	-50	14	-40
**MTL Subsystem**			
Ventral medial prefrontal cortex	0	26	-18
Posterior inferior parietal lobule	-44	-74	32
Retrosplenial cortex	-14	-52	8
Parahippocampal cortex	-28	-40	-12
Hippocampal formation	-22	-20	-26

Abbreviations: MNI, Montreal Neurological Institute coordinate; PCC, posterior cingulate cortex; aMPFC, anterior medial prefrontal cortex; dMPFC, dorsal medial prefrontal cortex ; MTL, medial temporal lobe

Reference

[1] Buckley CJ, Sherwin PF, Smith AP, Wolber J, Weick SM, Brooks DJ. Validation of an electronic image reader training programme for interpretation of [^{18}F] flutemetamol β-amyloid PET brain images. Nuclear medicine communications 2017;38(3):234-41
